# Painful and restricted hip due to myositis ossificans circumscripta of the pectineus muscle after pelvic fracture: A case report

**DOI:** 10.1097/MD.0000000000033694

**Published:** 2023-04-28

**Authors:** Qiushi Bai, Xiongfei Zou, Gang Yang, Yu Fan, Wenze Wang, Baozhong Zhang, Xiao Chang

**Affiliations:** a Department of Orthopaedics, Peking Union Medical College Hospital, Beijing, China; b Department of Pathology, Peking Union Medical College Hospital, Beijing, China.

**Keywords:** hip dysfunction, myositis ossificans circumscripta, pectineus muscle, pelvic fracture, surgery

## Abstract

**Rationale:**

Myositis ossificans circumscripta (MOC) is a rare disorder that causes heterotopic bone formation in soft tissues. It usually occurs after trauma and affects large muscles of the extremities. MOC of the pectineus muscle is extremely rare and has not been reported to be treated surgically.

**Patient concerns:**

A 52-year-old woman presented with left hip pain and dysfunction 4 months after a traffic accident that caused pelvic and humeral fractures and cerebral hemorrhage.

**Diagnoses:**

Radiological imaging revealed isolated ossification of the left pectineus muscle. The patient was diagnosed with MOC.

**Interventions:**

The patient underwent surgical resection of the ossified pectineus muscle followed by local radiation and medical therapy.

**Outcomes:**

At 12 months postoperatively, she was asymptomatic and had normal hip function. No recurrence was observed on radiography.

**Lessons:**

MOC of the pectineus muscle is a rare condition that can cause severe hip dysfunction. Surgical resection combined with radiation and anti-inflammatory drugs can be an effective treatment option for patients who do not respond to conservative management.

## 1. Introduction

Myositis ossificans (MO) is a rare disorder characterized by heterotopic bone formation in soft tissues and skeletal muscles.^[1]^ MO circumscripta (MOC), characterized by the ossification of a single muscle, is one of the 3 types of MO.^[1]^ Most MOC cases have been reported with a history of trauma, with few exceptions.^[2]^ More than 80% of lesions occur within the large muscles of the extremities, especially in the girdles and thighs. The affected muscles in published cases included: iliopsoas,^[3]^ triceps brachii,^[2]^ quadriceps femoris,^[4–6]^ biceps femoris,^[7,8]^ tibialis anterior,^[9]^ paravertebral,^[10]^ buccinators,^[11]^ masseter,^[12,13]^ etc. To the best of our knowledge, cases of MOC involving the pectineus muscle are rare,^[7]^ and no reported cases have been treated surgically.

We present the case of a 52-year-old woman with consistent and progressive pain in the unilateral groin and hip dysfunction after traffic trauma. Radiographs and computed tomography (CT) revealed ossification of the left pectineus muscle. After resection of the ossified muscle and postoperative radiation, as well as administration of anti-inflammatory drugs, she was in good condition and showed complete recovery of hip function.

## 2. Patient information

A 52-year-old woman was admitted to our hospital because of hip dysfunction and consistent and progressive pain in the left groin for 4 months after a traffic accident. In the accident, she suffered a coma for 2 days, and a minor cerebral hemorrhage was identified by a brain CT scan. She recovered without surgical intervention. She also sustained pelvic and left humeral diaphyseal fractures. Her left arm was immobilized for 2 months, and the displaced fracture of the humeral diaphysis healed with acceptable deformity and good function. However, she complained of persistent isolated pain in her left groin that worsened, and she could not move her left hip freely; consequently, she was unable to sit, stand, and walk 4 months after the accident. She had undergone a total hysterectomy and bilateral adnexectomy due to uterine prolapse when she was 49 years old and had no other significant medical history.

**Figure 1.**
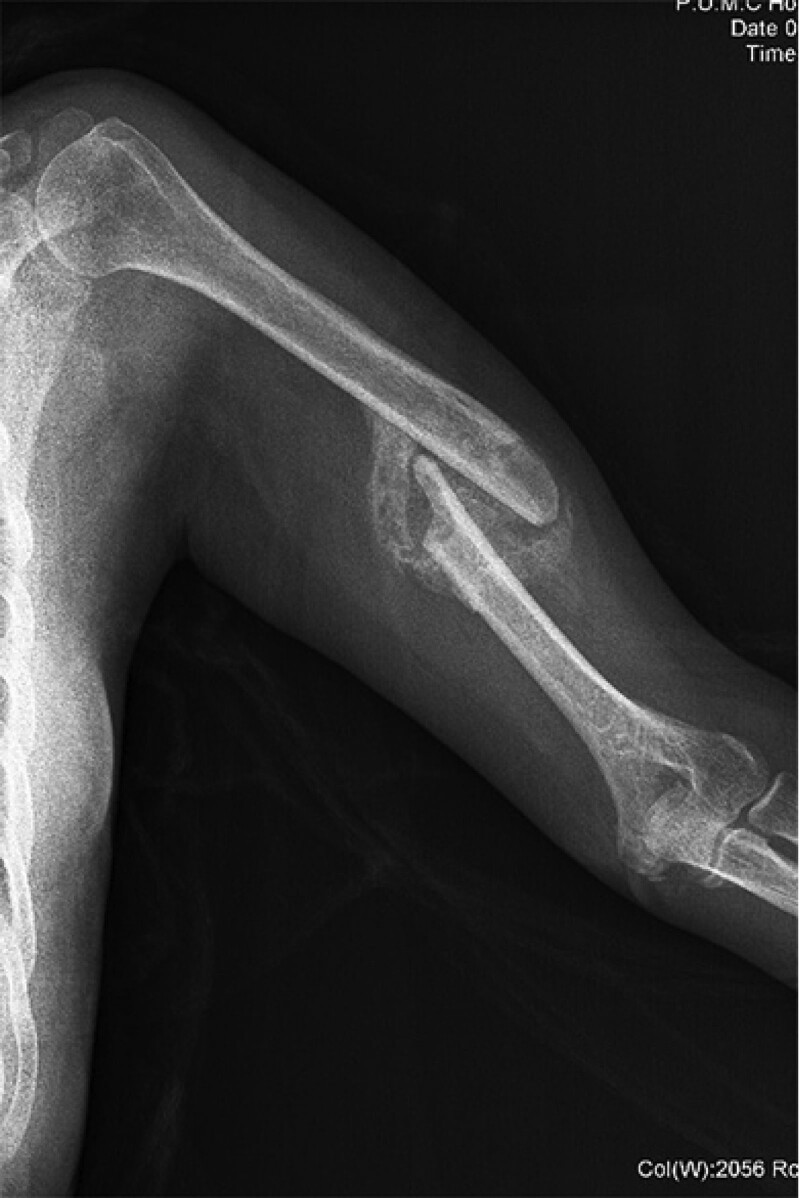
A radiograph of the left arm taken 4 mo after the accident reveals malunion of left humeral diaphysis with osseous bridge formation between displaced ends of fracture.

**Figure 2.**
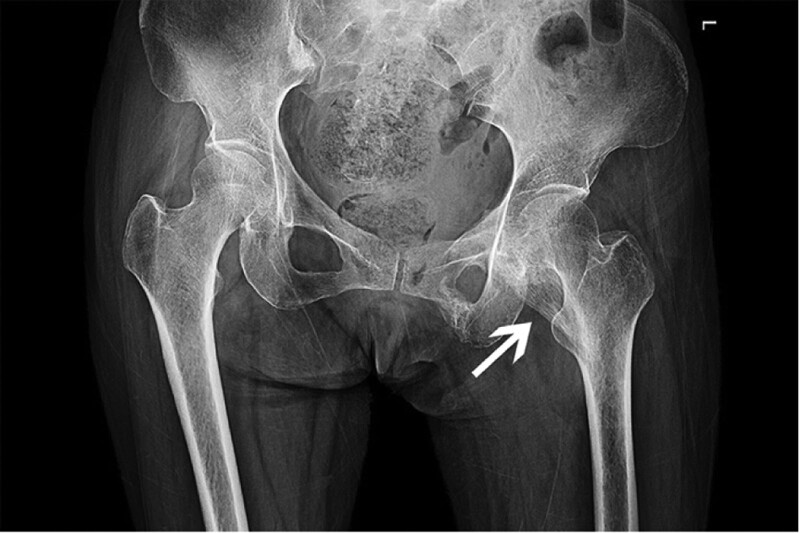
Radiograph of the pelvis taken 4 mo after the accident reveals an abnormal lesion with calcified radial fibers extending from the superior pubic ramus to the lesser trochanter (white arrow).

**Figure 3.**
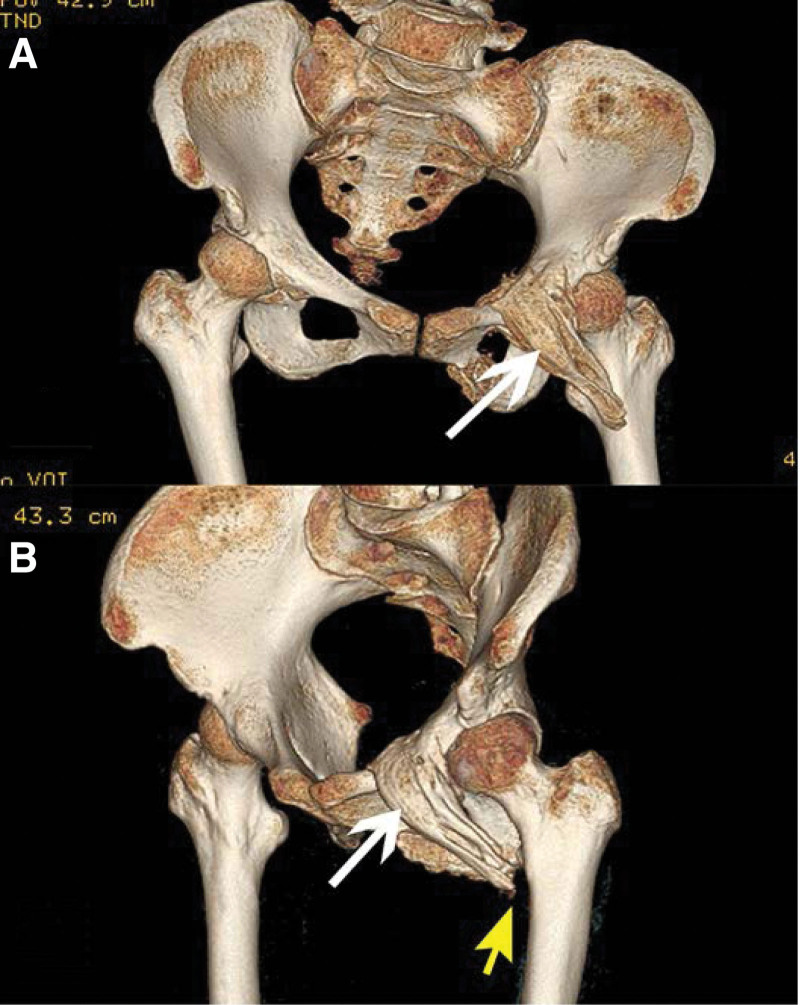
The 3-dimensional reconstruction CT views taken 4 mo after the accident. (A) Is the front view and (B) is the left oblique view. The views clearly show an osseous muscle-shaped lesion located between the superior pubic ramus to the lesser trochanter (white arrows) and a narrow space between the lesion and the lesser trochanter (yellow arrow). This suggested that the lesion may not be attached tightly to the femur. CT = computed tomography.

Physical examination and imaging data analysis after admission suggested MOC. Physical examination revealed the following: she was immobilized on the bed; there was a bony mass in her functional left mid arm; her left lower extremity was externally rotated and slightly abducted; tenderness was observed at her left groin; the range of the hip was reduced, and the passive range of motion was 30° of flexion, 0° of extension, 30° of abduction, and 0° of adduction. The radiograph of the left arm (Fig. 1) revealed a healed fracture of the humeral diaphysis with a deformity. A pelvic radiograph (Fig. 2) revealed healed fractures of the left superior and inferior pubic ramus, and an ossified lesion between the superior pubic ramus and lesser trochanter, which was made up of calcified muscle-fiber-like tissue with well-defined margins. Three-dimensional reconstructed CT images (Fig. 3) demonstrated that the left pectineus muscle was ossified. The patient was diagnosed with traumatic MOC of the pectineus muscle.

## 3. Surgical technique

**Figure 4.**
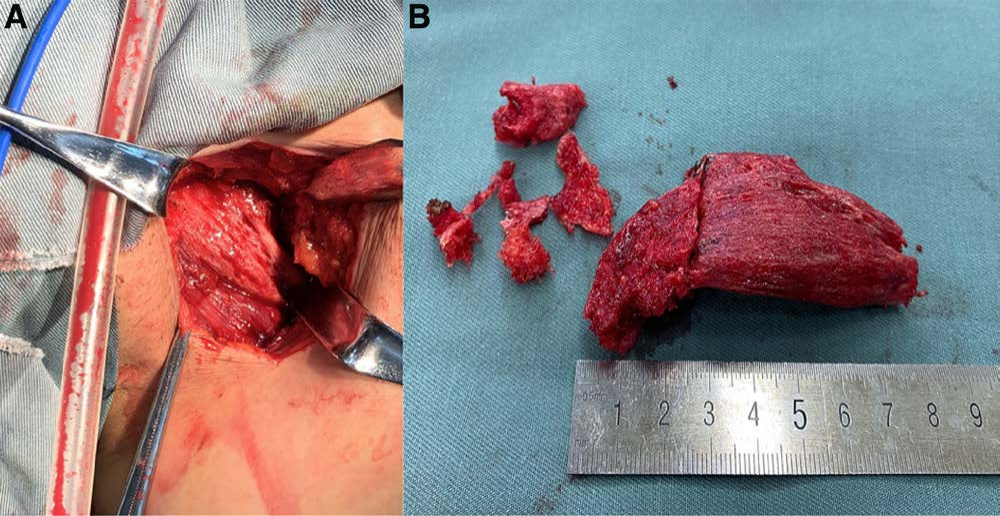
Through the groin incision, the left pectineus muscle was isolated (A). Operative findings demonstrated that most lateral part of the muscle was ossified, pale, and hard; the lesion was excised in one piece (B).

**Figure 5.**
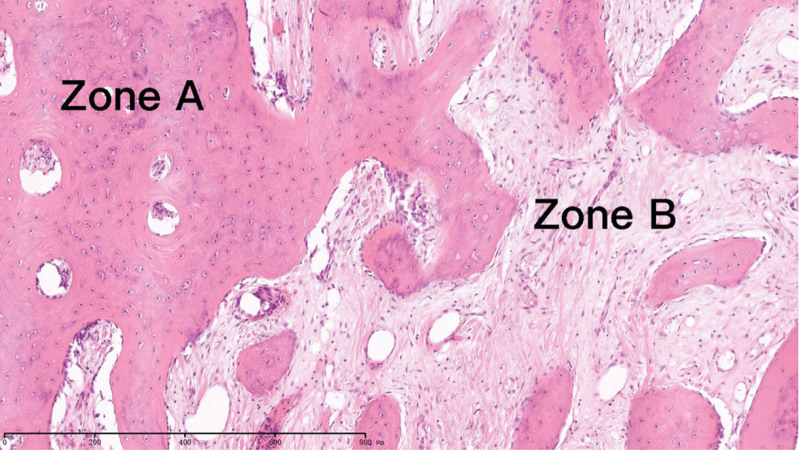
Histological examination demonstrating lamellar and trabecular bone formation (Zone A) with a peripheral surrounding fibrous capsule (Zone B) (hematoxylin-eosin staining, 50×).

**Figure 6.**
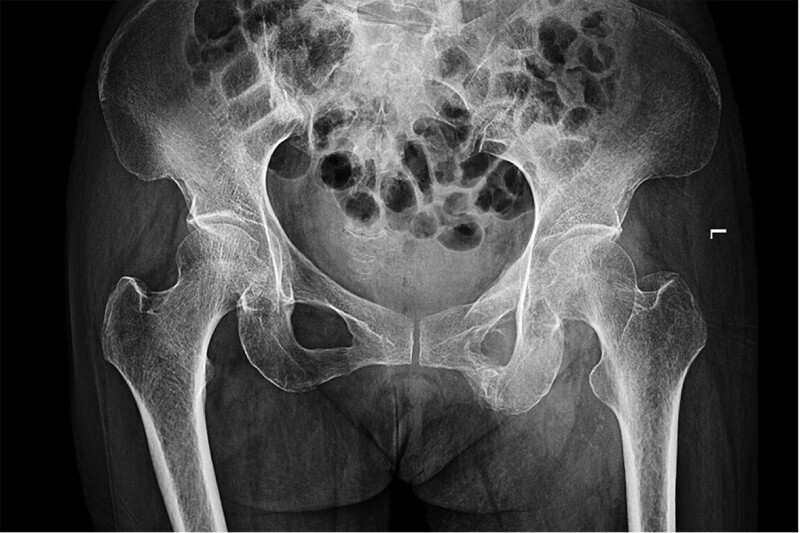
A radiograph of the pelvis taken postoperatively reveals complete resection of the lesion and no residual ossification.

The patient underwent ossified lesion resection. Although MOC is a self-limiting disease, her symptoms did not resolve spontaneously, and surgery was indicated. During the procedure, the pectineus muscle was exposed through an incision in the left groin (Fig. 4A); the texture of the muscle had toughened, including the origin of the superior pubic ramus. The origin was stripped using an osteotome, and the muscle was completely removed (Fig. 4B). MO was identified by histological analysis (Fig. 5). Postoperative radiography (Fig. 6) revealed no residual ossified lesions.

## 4. Postoperative course

After the surgery, the patient received 5 sessions of localized radiation therapy at a prescribed dose of 20 Gy; meanwhile, she received oral loxoprofen sodium (60 mg 3 times a day) for 4 weeks postoperatively.

**Figure 7.**
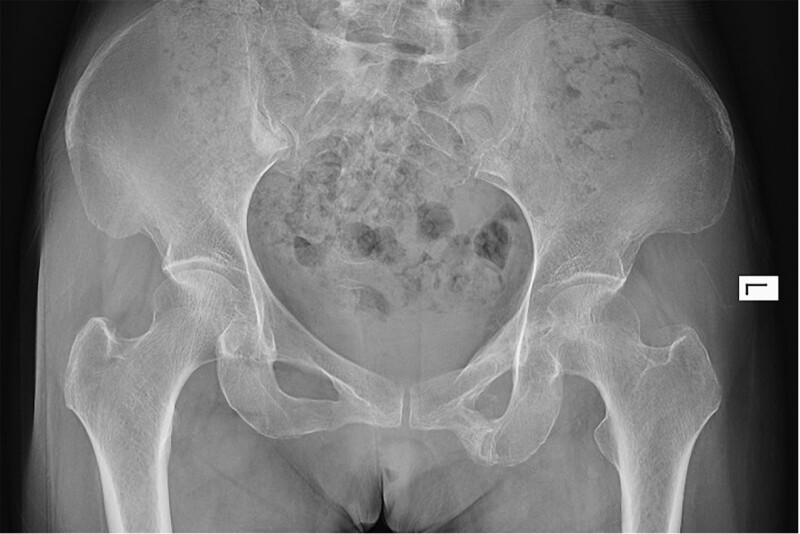
A radiograph of the pelvis taken 12 mo postoperatively reveals no recurrence or new ossified lesion formation.

At the 12-month follow-up, the patient had returned to normal life and work. The function of her left hip was good, and her Harris score was 90. No recurrence or new ossified lesions were identified on radiological examination (Fig. 7). The patient was satisfied with the results.

## 5. Discussion

MO is a rare disease characterized by heterotopic bone formation in soft tissues, mostly in skeletal muscle. Traumatic MO accounts for approximately 75% of cases, while the other 25% consists of neurogenic, congenital, and idiopathic MO.^[4,14]^ According to the hereditary, severity of diffuse heterotopic ossification, and underlying causes, MO can be divided into 3 types^[15]^: MOC, MO progressiva (fibrodysplasia ossificans progressive), and MO without a history of trauma or pseudo-malignant MO (non-traumatic MO). The last one includes cases associated with burns, polio, paraplegia, hemophilia, or infections.^[16]^

MOC is the most common form of MO with a history of trauma. It mainly affects the extremities and occasionally involves other sites, such as the temporal, abdominal, or paravertebral muscles.^[15]^ MOC can be observed in any age group but predominantly occurs in adolescents and young adults and is more common in men. MOC may develop from fracture, dislocation, or even minor soft tissue trauma. Inflammation induced by trauma may activate an osteogenic response, which initiates the release of periosteal cells and growth factors into the muscle, resulting in localized ossification.

In the present case, multiple factors may have contributed to the ossification of the pectineus muscle. The patient injured the pelvis, arm, and head; fractures of both pelvic and left humerus were treated conservatively, which made the immobilization on the bed stricter and longer. Immobilization is recognized as an important risk factor for heterotopic ossification or MO. It is known that after damage to the central nervous system, such as traumatic brain injury, stroke, or spinal cord injury, neurogenic heterotopic ossification may occur in periarticular muscles^[17]^; her traumatic cerebral hemorrhage may have aggravated the ossification process. neurogenic heterotopic ossification may involve systemic inflammatory changes in response to CNS injury, but the exact mechanism is still unknown.

No surgical case of MOC of the isolated pectineus muscle has been reported previously. Most published cases involve the thigh, girdle, arm, knee, and shoulder; the lesions develop from large muscles, and the calcification is usually extensive. One case of MOC that involved the pectineus muscle was treated conservatively.^[7]^ In the present case, the ossified lesion confined to the unilateral pectineus muscle may be explained by a coincidental broken site and minor limited fracture. The pectineus muscle was attached to the superior ramus of the pubis, where the patient’s pelvic fracture occurred, resulting in the formation of heterotopic bone in the pectineus muscle. Extensive ossification was avoided because the undisplaced superior ramus fractures had limited local trauma.

Imaging plays an important role in the diagnosis of MOC, especially when the lesion is calcified into the mature phase.^[16]^ The patient was referred to our hospital 4 months after the traffic accident, while it takes only 3 to 4 weeks for the muscle calcification to begin and become radiographically apparent. Her pelvic radiographs showed calcified lesions running parallel to the long axis of the pectineus muscle, which is a typical manifestation of mature MOC. However, in the early stages of MOC, because of the lack of ossified tissue, it may be difficult to diagnose this rare disorder. Occasionally, the periosteal reaction can be identified on plain radiography; soft tissue sarcomas associated with calcifications and extraskeletal osteosarcomas should be differentiated from the absence of a clinical presentation. CT can diagnose the condition earlier than radiography because it is more sensitive to soft tissue mass and calcifications. It can also delineate the zonal pattern of ossified muscle to determine MO and guide surgical resection. For difficult cases, additional imaging evaluations such as magnetic resonance imaging and bone scintigraphy may be necessary.

MOC is a benign, self-limiting, and self-resolving disease, and conservative treatment is preferred for the vast majority of cases.^[16]^ Surgical excision is indicated for intractable pain, dysfunction of the involved joint, and neurovascular structure compression caused by lesions.^[18]^ In our case, the lesion was present in front of the hip due to which the patient was unable to live a normal life. The ossified pectineus muscle was removed during the surgery. The pectineus muscle functions to flex and adduct the hip and internally rotate the thigh; however, these functions can be performed with other muscles such as the iliopsoas, adductor magnus, brevis, and longus. Removal of the pectineus muscle may have little effect on hip function, which was confirmed by the follow-up results of the case. For MOC, marginal excision is adequate; however, recurrence of the lesion has been reported. Radiation therapy can inhibit heterotopic ossification because of the sensitivity of osteogenic progenitors to radiation; in order to reduce the chance of recurrence, local radiation with a low dose is recommended in the early postoperative stage.^[19]^ Our patient underwent local radiation therapy 2 weeks after surgery, after the incision healed. Although bisphosphonates have been reported for MOC,^[5]^ the main medical therapy is nonsteroidal anti-inflammatory drugs (NSAIDs).^[18]^ Indomethacin is the most commonly used NSAIDs, and the recommended dose is 75 to 100 mg/d for 7 to 14 days postoperatively^[20]^; other NSAIDs are also effective. Because of its ease of availability and administration, loxoprofen sodium is the prescription option in our hospital.

## 6. Conclusion

In summary, even minor pelvic fractures may result in the MOC of adjacent muscles, especially in patients with heterotopic ossification risks, such as head injury, multiple fractures, and long-term bedrest. To our knowledge, MOC of the pectineus muscle is rare, and resection of the ossified pectineus muscle has not been previously reported. According to our experience, lesion resection combined with radiation therapy followed by oral NSAIDs may yield good results in symptomatic cases.

## Author contributions

**Conceptualization:** Yu Fan, Baozhong Zhang, Xiao Chang.

**Data curation:** Qiushi Bai, Xiongfei Zou, Gang Yang, Yu Fan, Wenze Wang.

**Methodology:** Xiao Chang.

**Resources:** Xiongfei Zou, Xiao Chang.

**Supervision:** Baozhong Zhang.

**Writing – original draft:** Qiushi Bai, Wenze Wang.

**Writing – review & editing:** Baozhong Zhang, Xiao Chang.
